# Ensemble machine learning-based scaling up of wetland carbon dynamics across the Southeastern U.S.

**DOI:** 10.1186/s13717-026-00755-9

**Published:** 2026-10-10

**Authors:** Ana Flavia Brancalion-Costa, Evan Farry, Latif Kalin, Mohamed M. Hantush, Zutao Ouyang

**Affiliations:** 1 College of Forestry, Wildlife and Environment, Auburn University, Auburn, AL 36849 USA; 2 U.S. EPA, Andrew W. Breidenbach Environmental Research Center, Cincinnati, OH 45268 USA; 3 School of Resource and Environmental Management, Simon Fraser University, Burnaby, Canada

**Keywords:** Wetland, Southeast United States, Machine learning, Carbon flux, Multi-model ensemble

## Abstract

**Background:**

Although the Southeastern United States (SE US) has been identified as a major terrestrial carbon sink, the balance of CO_2_ and CH_4_ fluxes from its wetlands remains poorly constrained due to strong environmental heterogeneity and limited regional-scale assessments. In this study, we used observations from 24 wetland sites located in the SE US, including freshwater and salt marshes, to train machine learning models (Random Forest, XGBoost, Support Vector Machine, Artificial Neural Network, and Partial Least Squares Regression) to predict monthly mean gross primary productivity (GPP), ecosystem respiration (RECO), net ecosystem exchange (NEE), and methane (CH_4_) fluxes.

**Results:**

Model performance measured in *r*^2^ for GPP ranged from 0.60 to 0.70, for RECO ranged from 0.44 to 0.56, for NEE ranged from 0.45 to 0.56, and for CH_4_ flux ranged from 0.33 to 0.37. Random Forest and XGBoost were the most consistent algorithms between fluxes. Feature importance analyses of these two algorithms indicated that CO_2_ flux predictions were primarily associated with vegetation and climatic variables, whereas CH_4_ predictions were more strongly associated with soil and hydrological properties. Using a performance-weighted multi-model ensemble (MME), we scaled up the predictions for the entire SE US from 2000 to 2020. Spatial patterns of productivity and respiration showed flux hotspots in the Everglades, the Mississippi River Valley, and the Gulf and East Coast. We also identified hotspots of CH_4_ emissions in coastal North Carolina. Slightly lower CH_4_ fluxes were observed between 2000–2007 in comparison to 2008–2020. Mean annual wetland carbon fluxes were 280 ± 38 Tg CO_2_-C for GPP, 233 ± 51 Tg CO_2_-C for RECO, − 20 ± 18 Tg CO_2_-C for NEE, and 5.7 ± 1.7 Tg CH_4_-C for CH_4_.

**Conclusions:**

Our results provide regional estimates of CO_2_ and CH_4_ fluxes that improve carbon budget assessments and support future wetland management in the SE US. By identifying the spatial and temporal variability of carbon uptake and emissions across the region, this study provides a foundation for improving regional carbon accounting and informing wetland conservation and management strategies.

**Supplementary Information:**

The online version contains supplementary material available at https://doi.org/10.1186/s13717-026-00755-9.

## Introduction

Reliable estimation of carbon dioxide (CO_2_) and methane (CH_4_) fluxes between the atmosphere and the biosphere is essential for understanding carbon-climate feedbacks and the biogeochemical processes that shape Earth’s climate system dynamics (Bonan 2008; IPCC 2007). Despite covering a relatively small area, wetland carbon fluxes represent a key component of the global carbon cycling (Zedler and Kercher 2005). In wetlands, carbon sequestration occurs through the uptake of atmospheric carbon by vegetation and its subsequent storage in biomass and organic soils. Consequently, wetlands can store large amounts of carbon over long periods of time, owing to their permanently or seasonally flooded conditions and adapted vegetation. North American wetlands contain approximately 220 Pg of carbon stored in their soils and vegetation pools (Bridgham et al. 2006), while total global wetlands carbon stocks are estimated to range between 520 and 710 Pg (Poulter et al. 2017).

Simultaneously, these ecosystems also serve as sources of non-CO_2_ greenhouse gases, particularly CH_4_, mostly due to anaerobic conditions. Wetlands are regarded as the largest natural source of CH_4_, with global bottom-up emissions estimated at ~ 149 (102–182) Tg of CH_4_ per year (Saunois et al. 2020).

Wetlands in subtropical and tropical regions, particularly in coastal zones, are major contributors to carbon exchange and storage. The Southeastern United States (SE US), encompassing the Atlantic and Gulf Coastal Plains, contains one of the highest densities of wetlands in North America (De Steven and Harrison 2022). At the regional scale, the SE US has been identified as one of the largest terrestrial carbon sinks in the United States (Schimel et al. 2000), while its coastal wetlands simultaneously represent a global hotspot for mangrove expansion (Osland et al. 2022). Yet, despite their substantial contribution to national and global carbon dynamics, a comprehensive spatiotemporal estimate of carbon fluxes across wetlands in the SE US remains lacking. Existing studies have either primarily focused on individual fluxes, such as CH_4_ (He et al. 2025), or combined CO_2_ and CH_4_ fluxes at a limited number of sites without spatial scaling up (Delaria et al. 2024), constraining our ability to assess spatial heterogeneity and emergent regional patterns. Currently, large uncertainties remain regarding the estimates of regional carbon flux over SE US (Gomez-Casanovas et al. 2020; Delaria et al. 2024).

Several key factors contribute to these large uncertainties from modeling regional carbon flux. First, the complex interactions at the interfaces of soil, water, and vegetation make the carbon exchange dynamics very complicated in wetland systems (Li et al. 2024; Zhang et al. 2002). A wide range of environmental drivers (e.g., water level, plant community, temperature, length of the growing season, soil properties) were identified as influencers of wetland carbon exchange dynamics (Charles et al. 2023; Ward 2020; Zhang et al. 2025). These drivers not only vary widely but also influence wetland carbon processes in nonlinear and asynchronous ways. As a result, it is challenging for current process-based models to accurately represent these complex and interacting processes. Secondly, the substantial spatial variability of wetland carbon dynamics (Wang et al. 2024), further increases the complexity of regional-scale biogeochemical modeling and highlights the limitations of oversimplified global estimates. Lastly and more importantly, the limited availability of observational flux measurements restricts the ability to constrain and benchmark wetland emission models, and specifically to improve the model’s ability to generalize across spatial heterogeneity.

To overcome some of these limitations, this study leverages machine learning (ML) models and carefully compiled observational datasets to model grid-based carbon fluxes across wetlands in the SE US. Eddy covariance (EC) towers and gas chambers are both widely used to measure carbon fluxes between terrestrial ecosystems and the atmosphere. EC towers provide quasi-continuous, multiyear measurements of CO_2_, CH_4_, water vapor, and energy fluxes over a relatively large footprint (Baldocchi et al. 2001). Gas chambers, in contrast, are portable and can be deployed more densely within a site often at biweekly or monthly intervals (Villa et al. 2021). Together, these approaches provide complementary spatial data coverage, and we thus compiled as many of both types of data as possible to enhance model training and constraint.

In recent years, data-driven approaches for predicting CO_2_ and CH_4_ fluxes have gained momentum, supported by the growing availability of environmental and flux observations as well as advanced ML algorithms. ML-based models offer several advantages, including the ability to capture complex, nonlinear relationships without requiring predefined hypotheses or initial conditions (Beer et al. 2010). However, their predictive performance can decline under conditions not represented in the training data or when the data size is small. ML models are particularly well suited for statistical generalization and can be efficiently applied to spatially gridded extrapolations (Bodesheim et al. 2018; Tramontana et al. 2016). A growing number of studies have applied these techniques to simulate gas exchange in wetlands (Abdul-Aziz et al. 2018; Ma et al. 2025; Mitra et al. 2024). Previous comparisons have frequently found stronger predictive performance of tree-based algorithms, such as random forest (RF) and extreme gradient boosting (XGB) (Shi et al. 2022; Gu et al. 2025; Abbasian and Jamili 2025). Their performance may partly reflect their ensemble structure and ability to accommodate missing data. Overall, these studies highlight the potential of ML for improving the prediction of wetland carbon fluxes, yet most focus on a single constituent, a limited set of algorithms, or a narrow ecosystem context.

The objectives of this study were to: (1) train and cross-validate a suite of ML models including RF, XGB, support vector machine (SVM), artificial neural network (ANN), and partial least square regression (PLSR) to predict gross primary productivity (GPP), ecosystem respiration (RECO), net ecosystem exchange (NEE, the balance between carbon uptake and release), and CH_4_ fluxes based on the collected observation data; and (2) upscale these fluxes spatially and temporally using gridded environmental predictors from 2000–2020. We hypothesized that (i) tree-based ensemble models (RF and XGB) would outperform the other ML approaches in predicting carbon fluxes, and (ii) the upscaled fluxes would exhibit distinct spatial and temporal patterns across the US SE in response to variations in environmental drivers.

The models were developed using a dataset comprising monthly observations from 24 wetland sites, including both freshwater wetlands and salt marshes, measured by EC towers and gas chambers. A total of 27 environmental predictors were selected for model training, validation and extrapolation using gridded satellite-derived data. By integrating both CH_4_ and CO_2_ fluxes, which are often treated separately in carbon accounting, we aim to provide a more complete understanding of carbon storage and greenhouse gas emissions from SE US wetlands. Our final model offers a scalable framework with potential applications in land management, greenhouse gas inventories, and future climate change scenario analyses.

## Methods

### Study region

**Fig. 1:**
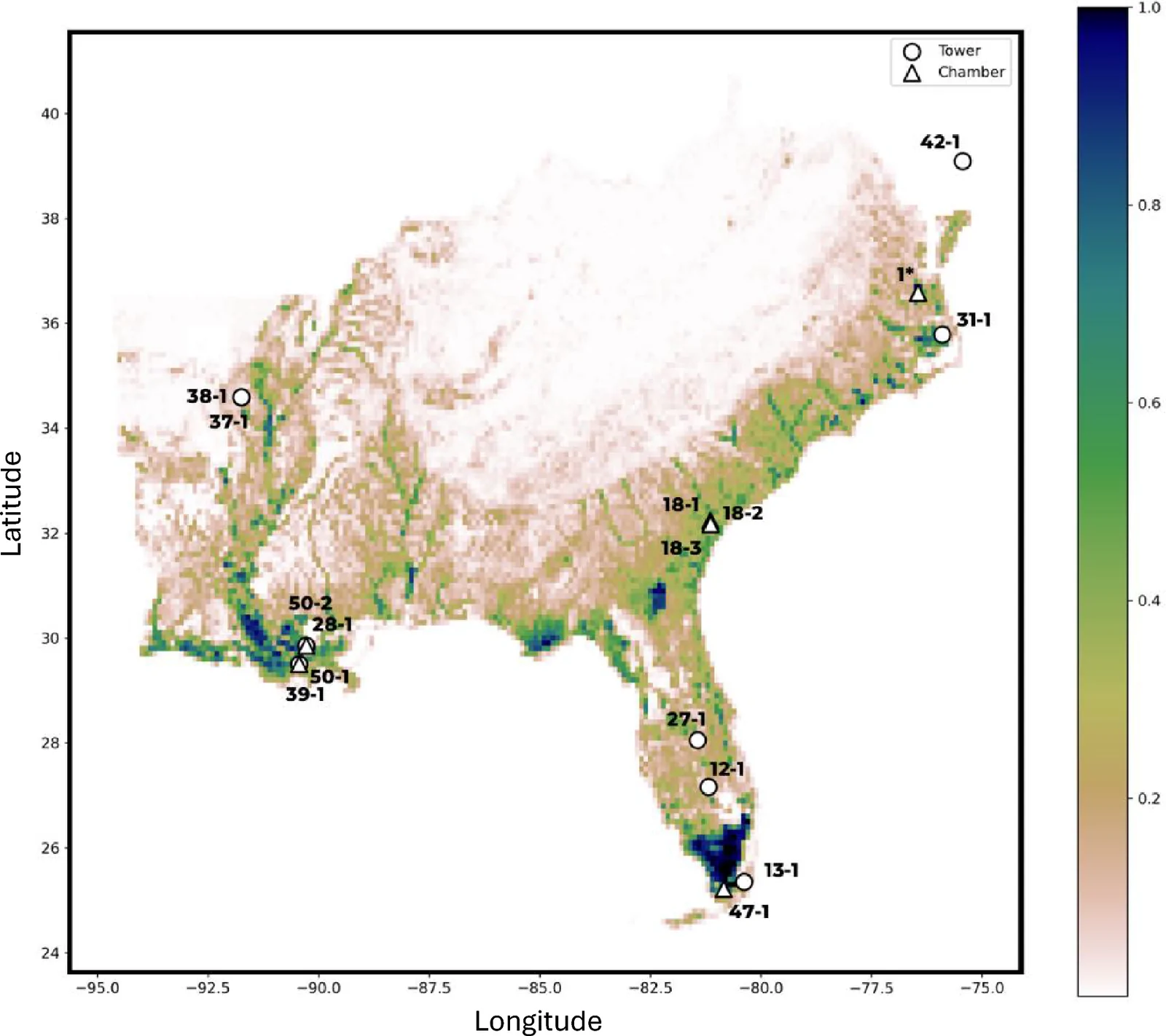
Study domain showing wetland fraction (0.1° × 0.1°) across the SE US and the locations of observation sites. Wetland fraction values range from 0 (no wetland) to 1 (entire grid cell is wetland). Circles indicate eddy covariance tower sites, and triangles indicate gas chamber sites. Site codes are labeled for reference

The modeling domain is the SE US, including the states of Alabama, Florida, Georgia, Mississippi, Louisiana, South Carolina, North Carolina, Tennessee, Kentucky, Virginia, West Virginia, and Arkansas (Fig. 1). The region’s climate is influenced by its broad latitudinal range, varied topography, and proximity to major water bodies. The northern and western portions of the region include prominent uplands such as the Appalachian Mountains (extending from Alabama to Virginia) and the Ozark Plateau in Arkansas. In contrast, low plateaus dominate from eastern Alabama to central Virginia, while extensive coastal plains span from Louisiana to southern Virginia in the south and east (Ingram et al. 2013).

The SE US is characterized by a humid subtropical climate, with warm, moist summers and more pronounced temperature gradients in winter. According to the updated Köppen-Geiger climate classification by Beck et al. (2023), most of the region is classified as temperate, without a dry season, and hot summers (“Cfa”). However, parts of southern Florida exhibit tropical rainforest (“Af”), monsoon (“Am”), and savanna (“Aw”) climates, and a small area between Virginia and Georgia falls under the cold, warm-summer classification (“Dfb”). Precipitation patterns are strongly influenced by topography and coastal proximity, with the highest precipitation occurring along the southern and eastern coasts (Kunkel et al. 2013). According to EPA Level I Ecoregions (CEC, 1997), most of the SE US falls within the Eastern Temperate Forest, with a smaller portion of southern Florida classified as Tropical Wet Forest.

The SE US distinctive topography and relatively abundant rainfall have resulted in extensive wetland formation greater than in any other region in the conterminous United States (Hefner and Brown 1984). However, wetland distribution is not uniform across the SE US. Florida, Louisiana, and South Carolina contain the highest densities of wetlands. Based on the U.S. Fish and Wildlife Service’s National Wetlands Inventory (NWI), freshwater forested/shrub wetlands represent the dominant wetland type in the region, accounting for approximately 78% of the total wetland area, followed by freshwater emergent wetlands (13%) and estuarine and marine wetlands (9%). Although representing a smaller proportion of the region’s wetlands, coastal wetlands include extensive tidal marshes along the Atlantic and Gulf of Mexico coasts, which are among the major areas of tidal marsh distribution globally (Worthington et al. 2024). Despite their extent, wetlands in the SE US have undergone substantial alteration due to agricultural expansion, industrial and urban developments over the past several decades (Hefner and Brown 1984).

### Data sources and preprocessing

#### Observational flux data

We compiled continuous and high-frequency carbon flux data from EC towers and discrete carbon flux measurements from static gas chambers, covering GPP (CO_2_ uptake), RECO (CO_2_ release), NEE (net CO_2_ release), and CH_4_ emissions (Brancalion-Costa et al. 2025a). Positive NEE values correspond to net carbon emissions, while negative values reflect net sequestration. Data were obtained through searches in scientific literature databases and dataset search engines, including Google Dataset Search, Google Scholar, and Web of Science. In total, we gathered data from 24 wetland sites (natural wetlands and rice paddies) across 13 different datasets. Of these, 14 sites used chamber-based measurements and 10 used EC towers (Table 1). To ensure consistency across methods, all flux observations were expressed as monthly mean flux rates, with GPP, RECO, and NEE reported in mg CO_2_-C m^−2^ hr^−1^ and CH_4_ emissions in µg CH_4_-C m^−2^ hr^−1^. Continuous high-frequency EC tower data were averaged into monthly averages, while chamber measurements, typically collected biweekly or monthly, were used to calculate or represent monthly mean flux rates. Reflecting the general wetland distribution in the SE US, 18 sites are located in freshwater wetlands, while 6 are in salt marshes or areas with a salinity gradient. The time span of the dataset ranges from October 2005 to December 2022, though the number of observations per site varies considerably. For example, the EC tower site US-NC4 in North Carolina includes 60 site-months of data from January 2012 to December 2016, while the EC towers US-HRA and US-HRC contribute only five months of data from the summers of 2015 and 2016. In total, the compiled dataset includes 318 site-months of NEE, 581 of RECO, 306 of GPP, and 458 of CH_4_.

**Table 1:** Summary of observational carbon flux sites used for model training and validation

Site	Site-month	Start year-month	Lat	Lon	State	Site ID	Salinity/Wetland type*	Method	Reference
1–1 1–2 1–3 1–4 1–5 1–6 1–7 1–8 1–9	20	2015-05	36.583	− 76.450	Virginia	GDSNWR	Freshwater/Forested	Chamber	Gutenberg et al. (2019)
12–1	31	2013-04	27.163	− 81.187	Florida	US-MAC	Freshwater/Emergent	Tower	Knox et al. (2021)
13–1	32	2020-05	25.354	− 80.381	Florida	US-EvM	Gradient/Emergent	Tower	Zhao et al. (2019)
18–1	24	2005-10	32.240	− 81.150	Georgia	–	Freshwater/Forested	Chamber	Krauss and Whitbeck (2012)
18–2	24	2005-10	32.180	− 81.140	Georgia	–	Gradient/Scrub-Shrub	Chamber	
18–3	24	2005-10	32.170	− 81.140	Georgia	–	Gradient/Emergent	Chamber	
27–1	44	2013-05	28.052	− 81.436	Florida	US-DPW	Freshwater/Forested	Tower	McNicol et al. (2023)
28–1	24	2011-12	29.859	− 90.287	Louisiana	US-LA2	Freshwater/Emergent	Tower	McNicol et al. (2023)
31–1	60	2012-01	35.788	− 75.904	North Carolina	US-NC4	Freshwater/Forested	Tower	McNicol et al. (2023)
37–1	2	2015-06	34.585	− 91.752	Arkansas	US-HRA	Freshwater/Forested	Tower	Runkle et al. (2018)
38–1	3	2015-06	34.589	− 91.752	Arkansas	US-HRC	Freshwater/Forested	Tower	Runkle et al. (2018)
39–1	4	2012-05	29.501	− 90.445	Louisiana	US-LA1	Gradient/Emergent	Tower	Krauss (2019)
42–1	36	2015-01	39.088	− 75.437	Delaware	US-Stj	Salt Marsh/Intertidal	Tower	Vázquez-Lule and Vargas (2021)
50–1	11	2012-03	29.859	− 90.287	Louisiana	Davis Pond	Freshwater/Emergent	Chamber	Krauss et al. (2016)
50–2	5	2012-03	29.501	− 90.445	Louisiana	Pointe-aux-Chenes	Freshwater/Emergent	Chamber	
51–1	81	2018-01	33.345	− 79.196	South Carolina	US-HB1	Sal Marsh/Intertidal	Tower	Forsythe et al. (2020)

*Vegetation classifications were derived from the National Wetlands Inventory (NWI). A description of each vegetation class is provided in Table S3

Although US-StJ is located in Delaware and lies slightly outside the SE US study domain, it was retained because it represents environmental conditions occurring near the northern boundary of the study region and improves the environmental coverage of the training dataset.

For sites reporting monthly observations, we retained the original values, while biweekly measurements were averaged to produce monthly estimates. When multiple chambers were deployed at the same site, their values were averaged to represent site-level fluxes. To ensure consistency across datasets, all flux measurements were converted to standardized units. EC tower data were obtained from AmeriFlux and FLUXNET networks. Some sites provide standardized, gap-filled monthly flux products through FLUXNET, while for others only half-hourly or daily data are available. When available, we used the preprocessed monthly FLUXNET products. For AmeriFlux sites and FLUXNET stations without monthly aggregates, we identified temporal gaps (any month with less than 40% data availability was excluded to reduce bias from incomplete records) and applied gap-filling procedures consistent with the FLUXNET methodology described by Pastorello et al. (2020).

We used RECO and GPP values as provided in the original datasets and did not perform additional partitioning of NEE into GPP and RECO components. Because EC towers directly measure NEE, we used these observed values rather than deriving NEE as the difference between GPP and RECO, a calculation that can introduce additional uncertainty. Tower- and chamber-based carbon flux measurements were treated as comparable for the purposes of model training (Al-Haj and Fulweiler 2020; Arias-Ortiz et al. 2024; Rosentreter et al. 2023). Additional preprocessing details for each dataset are described in the associated data release (Brancalion-Costa et al. 2025b).

#### Environmental predictors

A total of 27 environmental predictors were compiled to train the ML models, covering climate, vegetation, soil, and hydrological conditions known to influence carbon fluxes in wetland ecosystems (Arias-Ortiz et al. 2024; Knox et al. 2021; Yando et al. 2016). Climate variables included Atmospheric Pressure (PA), Precipitation (Prec), Air Temperature (${T}_{a}$) along with its monthly lags (${{T}_{a}}_{1}$, ${{T}_{a}}_{2}$, and ${{T}_{a}}_{3}$, representing 1, 2, and 3 months prior, respectively), Soil Temperature (${T}_{s}$) with its monthly lags (${{T}_{s}}_{1}$, ${{T}_{s}}_{2}$, and ${{T}_{s}}_{3}$), Surface Net Solar Radiation (${R}_{d}$) with its monthly lags (${{R}_{d}}_{1}$, ${{R}_{d}}_{2}$, and ${{R}_{d}}_{3}$), Vapor Pressure Deficit (VPD), and Wind Speed (WS). Hydrological variables included Water Level (WL) and Soil Water Content (SWC) with its monthly lags (${SWC}_{1}$, ${SWC}_{2}$, and ${SWC}_{3}$). For vegetation, we used the Enhanced Vegetation Index (EVI) as a proxy. Soil properties included bulk density (${\rho}_{b}$), Electric Conductance (Con), Organic Matter content (OM), and pH.

Con was used as a proxy for salinity, as higher conductance values generally reflect greater concentrations of dissolved ions and are characteristic of coastal and saline wetland environments. Lagged predictors were included to account for the influence of previous environmental conditions on current carbon fluxes, as wetland carbon dynamics often respond to environmental variability over periods of weeks to months (Knox et al. 2021; McNicol et al. 2023). WL was expressed relative to the soil surface, with positive values indicating standing water above the surface and negative values indicating water levels below the surface. For ${T}_{s}$ and its lagged variables, we used the soil temperature measurement closest to the surface. In ERA5-Land, this corresponded to *soil temperature level 1* (0–7 cm depth), while for the AmeriFlux/FLUXNET sites, we used soil temperature *profile 1* (0−2 cm depth) when available.

To represent wetland inundation conditions, we included a binary “Flooded Status” variable (1 for flooded, 0 for not flooded), which served as a proxy for sites without measured water level below ground. Seasonal variation was captured using a “Seasonality” index represented through a sinusoidal function fitted to the measurement month. This index ranged from values near 0 in the winter months to near 1 in the summer months, offering a continuous representation of seasonal progression.

These predictors were selected because they have been identified in previous studies as major environmental controls on wetland carbon cycling. Climate variables such as temperature, ${R}_{d}$, and VPD regulate photosynthesis, plant productivity, and microbial activity, thereby influencing GPP, RECO, and CH_4_ fluxes (Knox et al. 2021; Yuan et al. 2019). Hydrological variables, including WL and SWC, affect soil oxygen availability, redox conditions, and CH_4_ production pathways (Grant et al. 2012; Lu et al. 2017; Lieffers 1988), while PA can influence CH_4_ ebullition (Mattson and Likens 1990; Nadeau et al. 2013). Vegetation productivity is closely linked to carbon uptake and provides organic substrates that support respiration and methanogenesis (Kominoski et al. 2021). Soil properties such as OM, pH, ${\rho}_{b}$, and Con influence microbial processes and carbon storage capacity. The relative importance of these controls may vary among wetland types and environmental settings (Inglett et al. 2012; Chen et al. 2021; Davidson and Janssens 2006).

Whenever possible, environmental predictor values were extracted directly from the original flux datasets (EC towers and gas chambers). However, many datasets, particularly those derived from chamber measurements, lacked complete coverage of all variables. To address these gaps, additional data were integrated from gridded remote sensing and reanalysis datasets, including MODIS (Moderate Resolution Imaging Spectroradiometer), ERA5-land (Fifth-Generation Land Reanalysis, European Centre for Medium-Range Weather Forecasts), and SSURGO (Soil Survey Geographic Database, USDA), as detailed in Table S1. Soil property data were treated as time-invariant, with a single representative value assigned to each site for the entire study period. All time-dependent predictors were aggregated or extracted at monthly resolution to match the temporal scale of the flux measurements.

### Machine learning modeling framework

#### Model selection and training

To capture the complex and nonlinear relationships governing carbon fluxes in wetland ecosystems, we selected a broad set of supervised ML algorithms that represent a range of modeling approaches, from interpretable linear techniques to advanced ensemble and deep learning models.

PLSR was selected as a linear regression technique that incorporates dimensionality reduction by projecting both predictors and response variables into a shared space (Geladi and Kowalski 1986). This approach is particularly well suited for datasets with multicollinearity, as it fits the regression model in an orthogonal space defined by latent variables that capture the maximum covariance between inputs and targets. While PLSR offers advantages in terms of simplicity and interpretability, it may be limited in its ability to capture nonlinear relationships and interactions present in more complex datasets.

Based on its ability to model complex, nonlinear relationships in high-dimensional feature spaces, we employed SVM, a kernel-based, non-parametric model that identifies the optimal hyperplane for separating data by maximizing the margin between classes while minimizing classification/regression error (Vapnik et al. 1997). Through the use of kernel functions, SVM can model complex, nonlinear relationships in high-dimensional feature spaces. Although powerful, the model is sensitive to noise and outliers and typically requires input feature scaling for optimal performance. SVM is designed to balance generalization and accuracy, helping to prevent both overfitting and misclassification, particularly when the training data are limited or imbalanced.

We also implemented ANNs, which are nonlinear, non-parametric models inspired by the structure and function of biological neural networks (Dayhoff 1990). They consist of fully connected layers comprising input, hidden, and output nodes, linked by weighted connections and nonlinear activation functions. With appropriate tuning of architecture and hyperparameters, ANNs are capable of capturing complex, nonlinear relationships in data. However, they are prone to overfitting when trained on small datasets and often require large amounts of data to generalize well. Compared to linear models, ANNs have the potential to achieve lower errors and reduced bias, but they typically require longer training times and greater computational resources.

We also included RF, a bagging (bootstrap aggregating) ensemble learning algorithm that builds multiple decision trees to model nonlinear relationships while remaining robust to noise and overfitting (Breiman 2001). A decision tree is a flowchart-like structure composed of nodes, where each node represents a split based on an input feature. In the bagging process, each tree is trained on a bootstrapped subset of the data, and the final prediction is obtained by aggregating the outputs of all trees, typically through majority voting (classification) or averaging (regression). The strength and independence of individual trees are key to minimizing the overall generalization error.

To address missing values in the predictor dataset, we tested two imputation strategies. MissForest is a non-parametric algorithm that uses RFs to iteratively fill data gaps by predicting missing values based on the observed portion of the dataset (Stekhoven and Bühlmann 2012). MICE (Multiple Imputation by Chained Equations), in contrast, applies a series of regression models to impute missing values for each variable in turn (van Buuren and Groothuis-Oudshoorn 2011). We implemented a simple RF model as well as two additional pipelines that combined RF with MissForest and RF with MICE to evaluate the effect of imputation strategies on model performance.

For boosting-based modeling, we applied XGB (Extreme Gradient Boosting), an ensemble algorithm that constructs decision trees sequentially, with each new tree aiming to correct the errors made by the previous ones (Chen and Guestrin 2016). This boosting strategy combines multiple weak learners to form a strong predictive model. XGB includes built-in regularization to prevent overfitting, and it is optimized for performance through parallel computation, making it both efficient and scalable. A key advantage of XGB is its ability to handle missing values natively by automatically assigning a default direction for missing data at each tree node during training. To evaluate the impact of this feature, we tested two approaches: (1) removing records with data gaps prior to training, and (2) retaining the gaps and allowing XGB to handle them during training.

We also assessed predictor importance for both RF and XGB using their respective built-in feature importance metrics. Importance values were normalized to sum to one, with higher values indicating greater contributions to model predictions. Because correlated predictors, such as temperature variables and their temporal lags, may share importance, these values were interpreted as measures of predictive contribution rather than direct causal effects.

All ML models were implemented in Python using established open-source libraries. The RF, SVM, and PLSR models were implemented using the *scikit-learn* library (Pedregosa et al. 2011). XGB was developed using the *xgboost* package (Chen and Guestrin 2016), which provides an optimized implementation of gradient boosting algorithms. ANNs were constructed using the *TensorFlow* library (Abadi et al. 2016) with the *Keras* API for building and training deep learning models.

Hyperparameter tuning was performed using grid search approach, with the tested parameter ranges detailed in Table S2. In the grid search approach, predefined values for each hyperparameter were systematically evaluated by testing all possible combinations. When the optimal value identified during tuning coincided with the upper or lower bound of the tested range, we expanded the search interval (if reasonable), to ensure that the parameter space was adequately explored and that the optimum was not constrained by the initial limits.

All models were trained using a consistent leave-one-group-out cross-validation (LOGO-CV) strategy to assess model generalization and spatial extrapolation capabilities. During each iteration, one site was excluded from model training and reserved exclusively for validation, thereby testing the model’s ability to predict fluxes at previously unseen locations. Although this approach generally results in lower predictive performance than conventional random cross-validation, it provides a more realistic and rigorous assessment of model transferability (Roberts et al. 2017).

Model performance was evaluated using standard metrics, including the correlation-based coefficient of determination (*r*^2^), root mean squared error (RMSE), and mean absolute error (MAE). Overall model performance was calculated as the mean of the performance metrics obtained for each held-out site during the LOGO-CV procedure.

For SVM and ANN models, input features were standardized using *z*-score normalization (mean of 0 and a standard deviation of 1) via the *StandardScaler* from *scikit-learn* (Pedregosa et al. 2011), with scaling parameters computed exclusively from the training data to prevent data leakage. For the ANN models, we applied early stopping to prevent overfitting, using a validation split of 20% and a patience of 200 epochs with the best weights restored.

#### Multi-model ensemble modeling

To improve predictive accuracy and represent uncertainty associated with model selection, we developed a multi-model ensemble (MME) independently for each of the carbon flux variable (CH_4_, GPP, RECO, and NEE). Ensemble performance depends not only on the predictive accuracy of the individual models but also on the diversity of their predictions and errors (Krogh and Vedelsby 1994; Ortega et al. 2022). Therefore, combining algorithms with different structures and assumptions can provide a more comprehensive representation of model uncertainty than relying on a single algorithm or structurally similar models.

Because a separate MME was developed for each carbon flux, an exact mass balance between GPP, RECO and NEE is not guaranteed. Following model evaluation, the selected algorithms were retrained using the complete dataset (i.e., all monitoring sites) to maximize the information available for parameter estimation before spatial extrapolation. The predictive performance reported in this study corresponds to the independent LOGO-CV evaluation, whereas the final maps were generated using these models trained on the full dataset.

This strategy has been successfully applied in previous studies for predicting carbon fluxes in wetland ecosystems (Chen et al. 2024; Mitra et al. 2024). We constructed the ensembles by applying a weighted linear combination of our ML models, using the likelihood function (${L}_{k}$) shown in Eq. 1 for each carbon flux. The likelihood values were normalized by the sum of all model likelihoods, ensuring that the resulting ensemble weights summed to one.

1 $${L}_{k}=\mathrm{exp}\left({{{r}}_{k}^2}-\frac{{\mathrm{R}\mathrm{M}\mathrm{S}\mathrm{E}}_{k}}{\overline{\mathrm{R}\mathrm{M}\mathrm{S}\mathrm{E}} }\right)$$

where ${r}_{k}^{2}$ is the correlation-based coefficient of determination for model k, ${\mathrm{R}\mathrm{M}\mathrm{S}\mathrm{E}}_{k}$ is the root mean squared error of model k, and $\overline{\mathrm{R}\mathrm{M}\mathrm{S}\mathrm{E} }$ is the mean RMSE across all candidate models. This formulation assigns higher likelihoods to models with stronger predictive skill (higher ${r}^{2}$) and lower prediction errors (lower RMSE). The MME framework was applied uniformly across all carbon flux variables and time steps (2000 to 2020, monthly) and was used in all subsequent spatial scaling up analyses described in the following sections.

Because CH_4_ fluxes typically follow a skewed long-tailed distribution, CH_4_ models were additionally evaluated using empirical cumulative distribution functions (ECDFs) to assess their ability to reproduce the observed frequency distribution of CH_4_ fluxes.

### Regional estimation

To extrapolate site-level measurements into regional estimates, we used our MME to scale up carbon flux predictions across the SE US based on gridded environmental inputs. This included climate data from the ERA5-Land reanalysis product (Figure S1), a vegetation proxy from MODIS (EVI—Figure S2), and soil and hydrological properties (Figure S3 and Figure S4) from SSURGO.

ERA5-Land provides monthly averaged climate data at a native spatial resolution of 0.1° × 0.1° (~ 11 km × 11 km) from 1950 to the present. Despite its moderate resolution, it includes most of the key variables required by our ML models, enabling consistent input coverage. From ERA5-Land, we extracted monthly averages of ${T}_{a}$, ${T}_{s}$, ${R}_{d}$, SWC, including their 1–3-month lags. We also obtained PA and Prec, converting all variables to consistent units. WS was derived from the u- and v-wind components using Eq. 2.

2 $$WS=\sqrt{{u}^{2}+{v}^{2}}$$

Additionally, VPD was calculated using ${T}_{a}$ and ${T}_{dew}$ from ERA5-Land, following Eq. (5).

3 $${e}_{sat}=0.6108{e}^{\left(\frac{17.27{T}_{a}}{{T}_{a}+273.3}\right)}$$

4 $${e}_{a}=0.6108{e}^{\left(\frac{17.27Tdew}{Tdew+273.3}\right)}$$

5 $$VPD={e}_{sat}-{e}_{a}$$

where ${e}_{sat}$ is the saturation vapor pressure (kPa), ${e}_{a}$ is the actual vapor pressure (kPa), ${T}_{dew}$ is the dew point temperature (°C), ${T}_{a}$ is also measured in °C.

Vegetation data were obtained from the MODIS Terra Vegetation Indices Monthly L3 Global 1 km product (MOD13A3.061) (Didan 2021). The EVI values were regridded (Figure S2b) to the target resolution using a nearest-neighbor interpolation with the *xESMF* package in Python, ensuring consistent spatial alignment across all model inputs (Glauch et al. 2025; Zhuang et al. 2023).

Soil properties ${\rho}_{b}$, Con, OM and pH and the hydrological variable WL were obtained from SSURGO database. This dataset provides standardized, high-resolution soil information across the conterminous US. Soil variables were considered static, with one representative value per grid for the entire study period. To ensure consistency, each soil variable was first masked by wetland fraction maps. Values were spatially averaged across wetlands within each grid cell, producing one representative soil value per grid. The resulting fields were regridded with *xESMF* (Figure S4) to match the modeling grid, where carbon fluxes were subsequently computed). Figure S3 shows the WTD from SSURGO. For consistency throughout this study, WTD was converted to a WL convention, where positive values indicate standing water above the soil surface and negative values indicate the water table below the soil surface. In this representation, values > 0 denote flooded conditions, whereas values < 0 indicate the water table is below ground level. Similar to the soil properties, WL was treated as a static predictor with one representative value per grid cell throughout the study period. Although this simplification does not capture temporal fluctuations in water levels, no gridded dataset with adequate spatial coverage and temporal continuity across the SE US was available for the entire 2000–2020 study period.

The final spatial resolution of the scaling up was determined by the coarsest input dataset, ERA5-Land, with a native resolution of 0.1° × 0.1° (~ 11 km at the equator). Temporally, the analysis was constrained to the 2000–2020 period based on the availability of MODIS EVI data. The machine learning models predicted carbon fluxes per unit wetland area. To account for mixed land cover, these predictions were multiplied by the fraction of each grid cell classified as wetland in National Wetland Inventory (Fig. 1). Consequently, only the wetland portion of each grid cell contributed to the final carbon flux estimates. Only freshwater forested/shrub wetlands, freshwater emergent wetlands, and estuarine and marine wetlands were included in this analysis.

## Results

### Site-level modeling performance across fluxes

The scatter plots comparing predicted and observed GPP values are presented in Figure S5 (column i). For most models, predictions aligned well with observations. RF and XGB outperformed SVM, ANN, and PLSR across most performance metrics, specially RMSE, and MAE (Table 2). SVM exhibited the greatest scatter around the 1:1 line despite its reasonably good performance metrics. Furthermore, visual inspection of the scatter plot indicated very poor agreement between observed and predicted values for at least one site (Site 13–1; Figure S5c, column i). Given this inconsistency, SVM was excluded from the final MME for GPP. Among all carbon fluxes, GPP yielded the highest overall model performance, with all models achieving *r*^2^ values above 0.60.

**Table 2:** Model performance metrics for ML algorithms and final MME developed for carbon flux predictions in SE US

Performance metrics	RF	XGB	SVM	ANN	PLSR
GPP (mg CO_2_-C/m^2^/hr)					
*r*^2^	0.69	0.70	0.68	0.60	0.68
RMSE	88	99	190	157	117
MAE	76	88	171	142	104
L(k)	2.77	2.56	–	1.48	2.18
Model weight	0.31	0.28	–	0.16	0.24
RECO (mg CO_2_-C/m^2^/hr)					
*r*^2^	0.56	0.53	0.46	0.44	0.45
RMSE	110	107	113	130	136
MAE	84	82	97	103	121
L(k)	1.89	1.88	1.67	1.42	1.36
Model weight	0.23	0.23	0.20	0.17	0.17
NEE (mg CO_2_-C/m^2^/hr)					
*r*^2^	0.52	0.54	0.56	0.45	0.53
RMSE	37	37	45	60	41
MAE	30	30	38	50	34
L(k)	1.97	2.01	1.71	1.09	1.82
Model weight	0.23	0.23	0.20	0.13	0.21
CH_4_ (µg CH_4_-C/m^2^/hr)					
*r*^2^	0.35	0.33	0.35	0.33	0.37
RMSE	2920	2969	2829	2977	3105
MAE	2426	2413	2275	2492	2560
L(k)	1.44	1.39	–	1.38	–
Model weight	0.34	0.33	–	0.33	–

Results are shown for GPP (mg CO_2_-C m^−2^ hr^−1^), RECO (mg CO_2_-C m^−2^ hr^−1^), NEE (mg CO_2_-C m^−2^ hr^−1^), and CH_4_ fluxes (µg CH_4_C m^−2^ hr^−1^)

RF and XGB were also the top-performing models for RECO predictions, outperforming the other models across almost all evaluation metrics (Table 2). ANN and SVM showed moderate performance, with higher RMSE and MAE values. As shown in Figure S5 (column ii), PLSR displayed the greatest scatter relative to the 1:1 line, despite reasonably good performance metrics. Additionally, both RF and XGB slightly overestimated lower RECO values and underestimated higher fluxes.

From Table 2, NEE fluxes were best predicted by SVM, followed by XGB, PLSR, and RF. Although RF and XGB exhibited slightly lower *r*^2^ values than SVM and PLSR, they achieved lower RMSE values. ANN demonstrated moderate performance overall. As shown in Figure S5 (column iii), the predictions displayed larger spread relative to observations, particularly for SVM and ANN, which tended to overpredict higher flux values.

For CH_4_ fluxes, PLSR yielded the best predictions, although their performance was lower compared to the CO_2_ flux predictions (Table 2). This pattern is likely related to the skewed distribution of CH_4_ fluxes, where extreme emission events are relatively rare and therefore difficult for ML models to learn since most ML algorithms tend to predict toward the mean and have difficulty reproducing rare extremes.

As shown in Figure S5 (column iv), the scatterplots revealed an unusual distribution of observed and predicted values for the SVM model. Further evaluation using the ECDF showed that neither SVM nor PLSR adequately reproduced the highly skewed distribution of CH_4_ fluxes. Consequently, both models were excluded from the final CH_4_ MME. The ECDFs of the final ensemble (Figure S6a), as well as those of RF (Figure S6b), XGB (Figure S6c), and ANN (Figure S6e), successfully captured the overall distribution of CH_4_ fluxes, although the highest emission values remained underestimated.

Feature importance analysis showed that EVI, seasonality, and ${R}_{d}$ were among the most influential predictors of GPP in both the RF and XGB models, highlighting the importance of vegetation and climatic conditions in predicting GPP (Figure S7). For RECO, the two algorithms identified similar important predictors, with EVI, VPD, and OM ranking among the five most important predictors in both models. For NEE, ${R}_{d}$, seasonality, ${T}_{a}$, and EVI ranked among the five most important predictors in both models, although ${T}_{s}$ was the most important predictor in XGB. For CH_4_, WS, OM, and Con were important predictors in both models, while pH was particularly important in RF and ${\rho}_{b}$ in XGB. Despite some differences in predictor rankings, RF and XGB models identified broadly similar groups of environmental predictors.

### Regional predictions

**Fig. 2:**
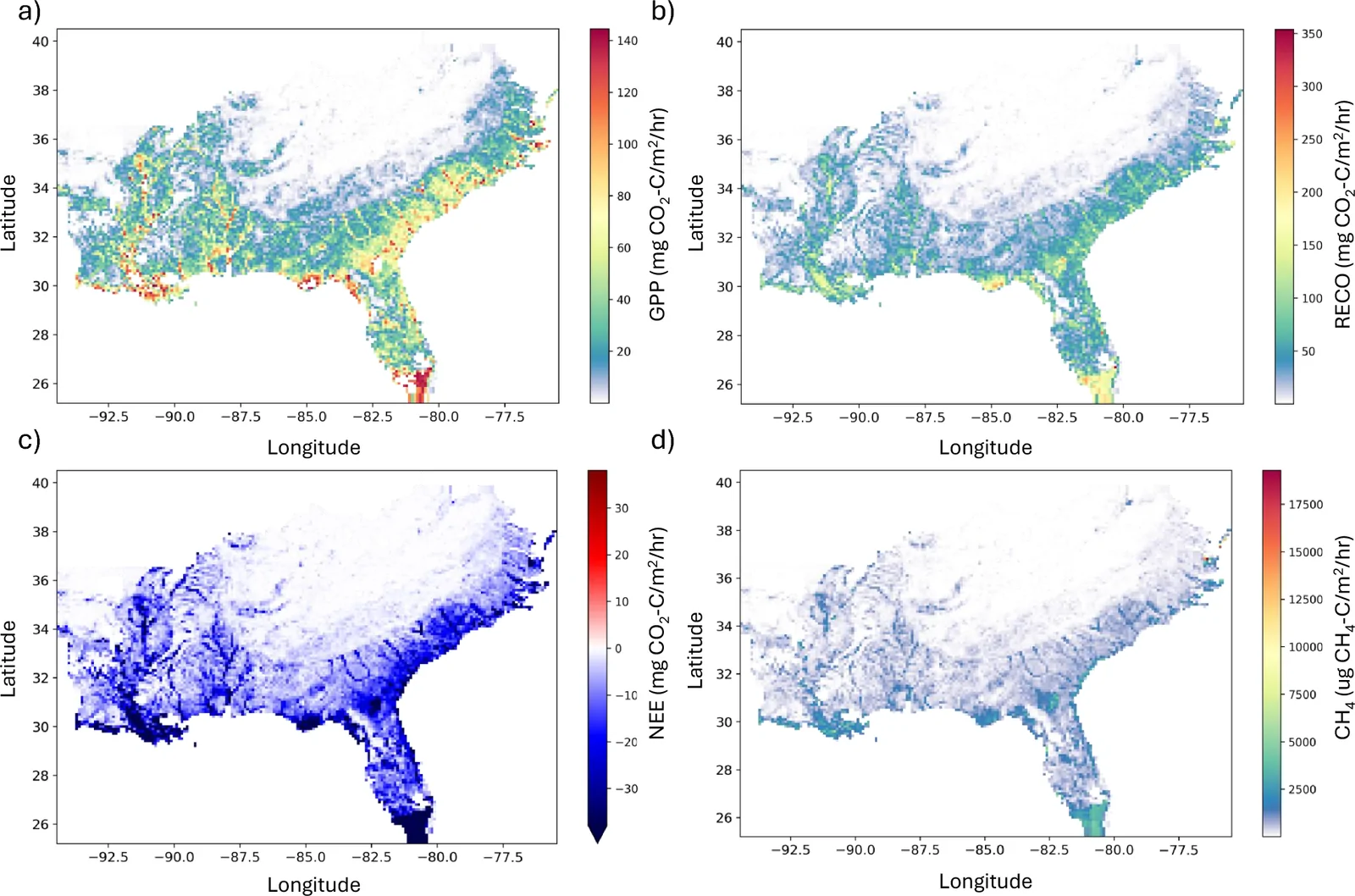
Average MME predictions of wetland carbon fluxes across the SE US for the period 2000–2020. Maps show spatial distributions of: **a** GPP (mg CO_2_-C m^−2^ hr^−1^), **b** RECO (mg CO_2_-C m^−2^ hr^−1^), **c** NEE (mg CO_2_-C m^−2^ hr^−1^), and **d** CH_4_ (µg CH_4_-C m^−2^ hr^−1^). Values represent the average of monthly ensemble outputs, weighted by wetland fraction in each grid cell. Negative values of NEE indicate net CO_2_ uptake

Figure 2 presents the predicted long-term averages of the scaled-up fluxes, obtained by multiplying model predictions by the wetland fraction, thus reflecting the estimated flux from wetland-covered areas only. The maps display the average flux for the study period (2000–2020) for GPP (a), RECO (b), NEE (c), and CH_4_ (d). MME predictions were generated for each grid cell (0.1° × 0.1°).

From Fig. 2a and b, GPP and RECO showed similar spatial patterns, with higher fluxes along the Gulf and Atlantic Coasts and the Mississippi River corridor. Both GPP and RECO fluxes reached maximum average values around 250 mg CO_2_-C/m^2^/hr. From Fig. 2c, NEE displayed also similar long-term hotspots of CO_2_ sequestration (negative NEE) along the Coasts and the Mississippi River corridor. Average NEE fluxes ranged from approximately 0 to –10 mg CO_2_-C/m^2^/hr. CH_4_ emissions exhibited a broadly similar spatial pattern, with elevated fluxes across Florida and several high-emission grid cells near the eastern edge of the domain (Fig. 2d). Maximum CH_4_ fluxes are around 10,000 µg CH_4_-C/m^2^/hr.

For GPP, the time-averaged predictions from the individual ML models were generally consistent across models (Figure S8). Among the models, ANN predicted the highest productivity hotspot, while RF and XGB produced very similar spatial patterns and magnitudes. Similar to GPP, the time-averaged RECO predictions were highly consistent across models (Figure S9). As observed for GPP, ANN predicted the highest CO_2_ emission hotspots, whereas the remaining models produced comparable spatial patterns.

The largest differences in time-averaged predictions among the ML models were observed for wetland-weighted NEE (Figure S10). RF, XGB, and particularly ANN predominantly predicted negative NEE values, indicating stronger net CO_2_ uptake. In contrast, SVM and PLSR produced a broader range of predictions, including areas with positive NEE values (net CO_2_ emissions) as well as regions with CO_2_ uptake. Despite these differences, all models consistently identified the strongest long-term CO_2_ uptake along the Gulf and Atlantic coastal plains, particularly in the Mississippi Alluvial and Coastal Plains. In contrast, wetlands in the Appalachian region generally exhibited weaker CO_2_ uptake or even net emissions (SVM and PLSR).

Similar to NEE, time-averaged CH_4_ predictions showed more variability in magnitude across the wetland-scaled models (Figure S11). RF, XGB, and ANN predicted the highest CH_4_ fluxes, whereas SVM consistently produced the lowest values. PLSR predicted intermediate fluxes, although its spatial patterns were generally consistent with those of RF and XGB. Overall, RF and XGB produced similar regional CH_4_ distributions, with elevated emissions concentrated along the Gulf Coast, coastal wetlands, and southern Florida, while lower emissions were predicted in the Appalachian region and inland wetland.

### Temporal dynamics of regional carbon fluxes

#### Seasonal cycles

**Fig. 3:**
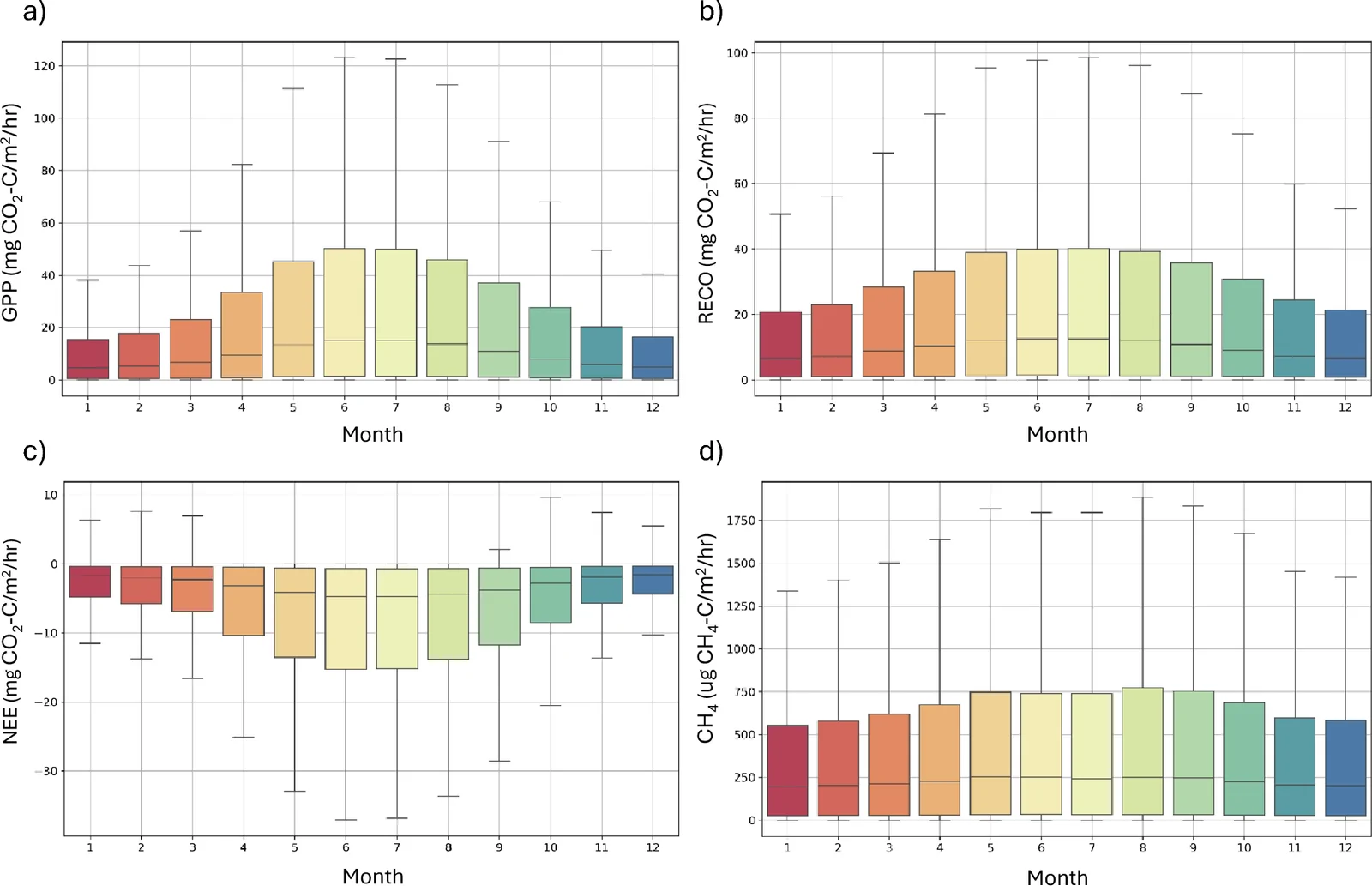
Monthly distributions of ensemble-predicted wetland carbon fluxes across the SE US for the period 2000–2020. Boxplots represent the monthly variability of: **a** GPP (mg CO_2_-C m^−2^ hr^−1^), **b** RECO (mg CO_2_-C m^−2^ hr^−1^), **c** NEE (mg CO_2_-C m^−2^ hr^−1^), and **d** CH_4_ (µg CH_4_-C m^−2^ hr^−1^)

All carbon fluxes exhibited seasonal patterns throughout the study period. Figure 3a shows monthly boxplots of GPP average predictions from the regional ensemble model. Both the median values and interquartile ranges are higher during the summer months, indicating greater flux magnitude and variability. This pattern is further supported by Figure S12, which compares time-averaged ensemble predictions for summer (June–July–August, JJA) and winter months (December–January–February, DJF), showing a substantial increase in GPP fluxes during summer. GPP shows pronounced hotspots of CO_2_ uptake during summer, particularly in the Mississippi River Delta, the Everglades in Florida, the Mobile Delta in Alabama, and along the Gulf and Atlantic coasts. A notable hotspot also appears in southern Georgia and northern Florida, corresponding to the Okefenokee Swamp, a highly productive wetland characterized by abundant vegetation, long growing seasons, and stable water levels in summer.

RECO displays a similar seasonal pattern to GPP (Fig. 3b, Figure S13), although with smaller seasonal variability. The summer respiration hotspots are present, especially around the Alabama-Mobile delta and the Everglades, but less pronounced. Larger interquartile ranges in summer months indicate greater spatial variability during the growing season.

Seasonality is also evident in NEE dynamics (Fig. 3c). Figure S14 shows greater CO_2_ sequestration during the summer months, particularly in humid regions near large rivers, and along the Gulf and Eastern coasts. The Everglades in South Florida and the Mississippi Valley consistently exhibited greater net CO_2_ uptake than other regions throughout the year. Figure S14 also highlights a shift toward net CO_2_ emissions during the winter months, although these emissions were of smaller magnitude than the net CO_2_ uptake observed during summer.

Monthly averages of CH_4_ fluxes across the SE U.S. also exhibited seasonal variation (Fig. 3d, Figure S15). Higher CH_4_ emissions during summer were concentrated near major wetland complexes, including the Everglades, coastal Louisiana, and parts of coastal North Carolina.

#### Interannual variability

**Fig. 4:**
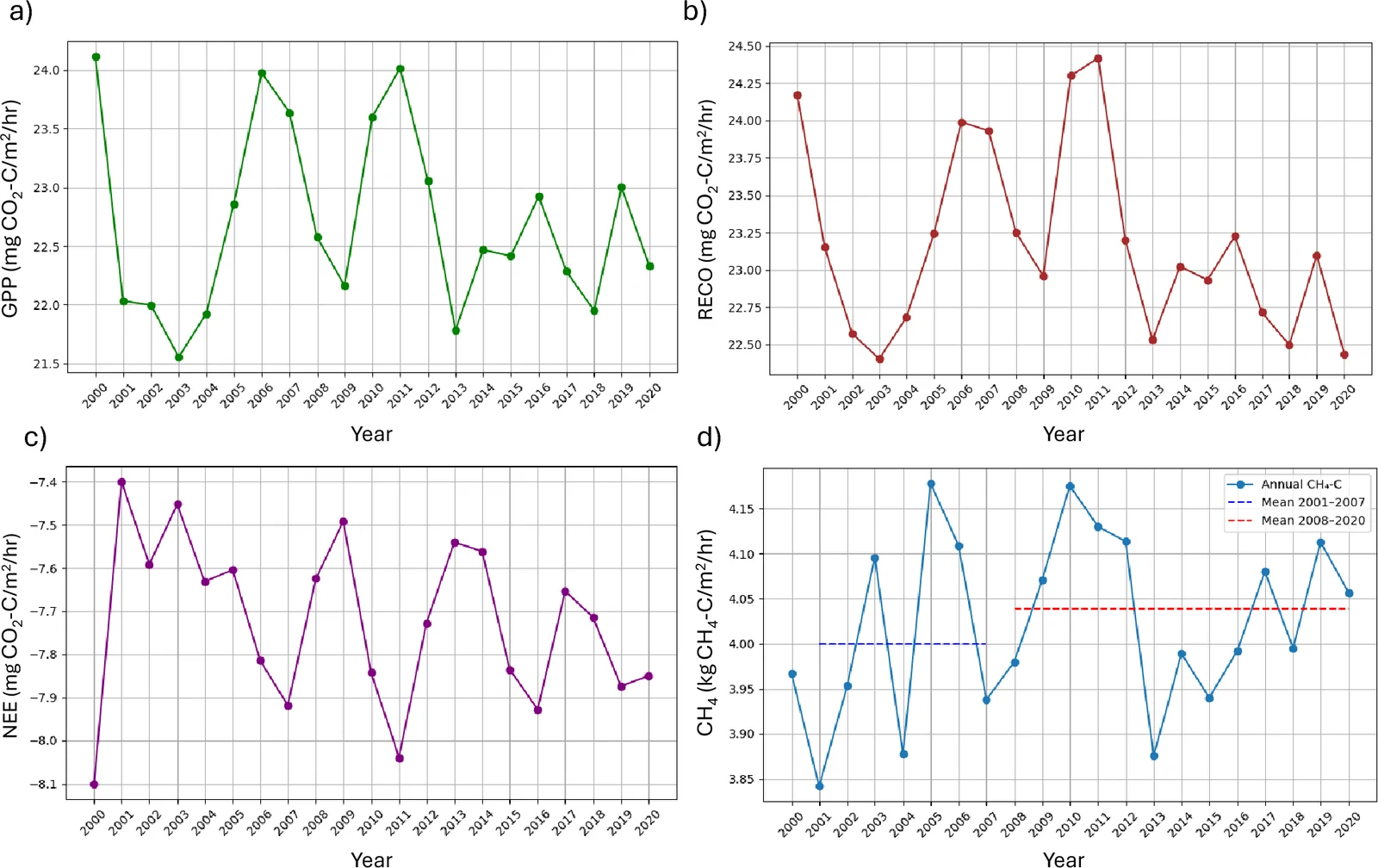
Annual average of monthly values of MME-predicted wetland carbon fluxes across the SE US from 2000 to 2020. Panels show yearly trends in: **a** GPP (mg CO_2_-C m^−2^ hr^−1^), **b** RECO (mg CO_2_-C m^−2^ hr^−1^), **c** NEE (mg CO_2_-C m^−2^ hr^−1^), and **d** CH_4_ (flux density sum, kg CH_4_-C m^−2^ hr^−1^). In panel **d**, horizontal dashed lines represent the average CH_4_ emissions for two distinct periods: 2001–2007 (blue) and 2008–2020 (red), highlighting an overall increase in mean CH_4_ fluxes after 2007. The gray dashed line in each panel represents the long-term mean (2000–2020)

The annual average of monthly carbon fluxes predicted by MME across the SE US exhibited year-to-year variability (Fig. 4). GPP and RECO followed similar increasing and decreasing trends in the same years, which reflects a strong correlation between the two variables. In contrast, NEE showed the opposite pattern, rising when GPP and RECO declined, and decreasing when they increased. This is consistent with its role as the net balance between carbon uptake and release. CH_4_ fluxes displayed interannual variability, although with a slight increasing trend over the period 2000–2020. The year 2000 was omitted from the annual plots because the upscaled dataset begins in February 2000, and including that year would bias the annual average due to the missing January data.

The lowest average annual estimated fluxes of GPP and RECO occurred in 2003, along with the second-highest peak in NEE. Figure S16 shows that 2003 was characterized by the second-largest negative anomalies in both ${T}_{a}$ and ${T}_{s}$, as well as the most negative anomaly in VPD. In contrast, SWC and precipitation exhibited moderately positive anomalies during that year, which was reported as one of the wettest of the past decades (Chan and Misra 2010). High NEE value (i.e., small negative value) suggests a reduced carbon sink capacity in wetlands during this period. When combined with elevated CH_4_ emissions, 2003 may represent a temporary hotspot of regional carbon release.

In 2013, fluxes of GPP, RECO, and CH_4_ were all relatively low, as well as ${T}_{a}$, ${T}_{s}$, VPD, and ${R}_{d}$ anomalies (Figure S16). Although no major precipitation extremes were recorded that year, it was characterized by lower temperatures, which may explain the reduced flux activity.

The largest peaks in CH_4_ emissions occurred in 2005 and 2019, while the highest fluxes of GPP and RECO were observed in the following years, 2006 and 2011. This suggests a possible lag between the drivers of CH_4_ emissions and CO_2_ related processes such as productivity and respiration. While CH_4_ responded more immediately, likely influenced by hydrological conditions, GPP and RECO may have taken longer to respond to the same environmental drivers, such as soil moisture or temperature anomalies following dry periods. The years with lower CH_4_ emissions (2001, 2004, and 2013) generally coincided with negative anomalies in ${T}_{a}$, ${T}_{s}$, VPD, and ${R}_{d}$, while precipitation anomalies were positive during these years.

When the predicted fluxes were multiplied by the actual wetland area within each grid cell and aggregated annually, the average estimated GPP was 280 ± 38 Tg CO_2_-C per year. Estimated RECO was 233 ± 51 Tg CO_2_-C per year, while NEE resulted in a net carbon uptake of − 20 ± 18 Tg CO_2_-C per year. For CH_4_, the estimated emissions were 5.7 ± 1.7 Tg CH_4_-C per year. These values represent the annual average carbon fluxes from wetlands across the Southeastern U.S. over the study period.

## Discussion

### Comparison of model performances across fluxes

Overall, and particularly for CO_2_ fluxes, our set of ML models performed well in predicting carbon fluxes at the observation sites. Similar sets of ML models have been successfully applied in other studies using flux tower measurements (Xu et al. 2018). Across all flux models, the ensemble models RF and XGB consistently outperformed the other non-ensemble algorithms. Both models incorporate mechanisms for handling missing data, which may have contributed to their ability to better model the nonlinear and complex dynamics associated with wetland carbon processes. For instance, He et al. (2025) also found that RF performed well for simulating CH_4_ dynamics in the SE US.

PLSR showed lower performance for RECO and CH_4_ predictions. Because this algorithm is limited in its ability to capture nonlinear relationships, these results suggest that nonlinear interactions between environmental drivers and carbon fluxes play an important role in wetland carbon dynamics. The SVM model also exhibited relatively poor performance, especially for RECO and CH_4_ fluxes, which is consistent with its known sensitivity to noise and outliers. This limitation may be particularly relevant for CH_4_, as the ECDF analysis showed that all models struggled to reproduce the highest emission events.

Among the fluxes, GPP had the highest model performance scores, followed by NEE and RECO with similar results, and CH_4_ with the lowest. This performance hierarchy aligns with findings from other studies, which often report stronger predictive capacity for GPP (Tramontana et al. 2016). The models for CO_2_ fluxes (particularly GPP and RECO) having higher accuracy than CH_4_ fluxes can be attributed to the strong and well-documented relationships between CO_2_ fluxes and climate drivers such as radiation and temperature, which were included as predictors in our models. In contrast, CH_4_ fluxes are known to exhibit greater complexity and nonlinearity, including lagged effects and high spatial variability (Delwiche et al. 2021; Knox et al. 2021; McNicol et al. 2023), which make them more challenging to predict. We should also acknowledge that, even with a relatively large set of predictors (27 environmental variables), some key drivers of CH_4_ emissions may still be missing. For example, CH_4_ fluxes are known to be strongly influenced by water level dynamics and salinity, and our use of a static WL predictor and Con as a proxy for wetland salinity may limit the model’s ability to capture temporal variability in wetland CH_4_ emissions. Additionally, although EVI was included as a proxy for vegetation density, previous studies have shown that vegetation community composition can exert a strong influence on CH_4_ emissions in both tidal (Mueller et al. 2020) and non-tidal wetlands (Bhullar et al. 2014; Stewart et al. 2024). Future studies would benefit from incorporating spatially explicit vegetation community data.

The ECDF analysis (Figure S6) complemented conventional performance metrics such as *r*^2^, RMSE, and MAE by providing additional insight into model performance for highly skewed, long-tailed flux distributions. RF, XGB, and ANN reasonably reproduced the overall skewed distribution of observed CH_4_ fluxes, although none of the models fully captured the highest emissions. In contrast, PLSR poorly reproduced the observed CH_4_ distribution, exhibiting a compressed range of predicted values and a tendency to underestimate the variability in CH_4_ fluxes. Similarly, SVM showed a poor representation of the observed CH_4_ distribution, with little variability in its predicted values.

The inability of the models to reproduce the largest CH_4_ emissions highlights an important limitation of the upscaling approach, as hotspot emissions may be underestimated in the final maps. Consequently, regional CH_4_ emissions derived from the ensemble should be interpreted with caution, particularly in areas where extreme emission events are likely to occur.

RF and XGB can generate predictions when some predictor values are missing because of their tree-based structure, whereas SVM, ANN, and PLSR require complete predictor data. Consequently, areas with missing predictor values resulted in gaps in the predictions of SVM, ANN, and PLSR, while RF and XGB maintained more complete spatial coverage.

### Spatial patterns in scaled-up fluxes

The map of GPP predicted by MME and weighted by wetland fraction revealed productivity hotspots in the lower latitudes of the SE US, particularly in South Florida, the Gulf Coast, and the Mississippi River Delta (Fig. 2). These regions are well known for receiving higher solar radiation, experiencing warmer temperatures, and supporting longer growing seasons, factors that are likely key drivers of elevated GPP (Han et al. 2014; Wei et al. 2022). The Everglades in Florida, in particular, are recognized for their intense productivity and dense wetland vegetation (Malone et al. 2022). Although Song et al. (2013) estimated GPP across the entire SE US, we compared our results specifically with their estimates for the Everglades, which ranged between 0.8 and 1.2 kg C/m^2^/yr around 2000 (equivalent to 91–137 mg CO_2_-C/m^2^/hr). These estimates closely align with our 2000–2020 mean (120–140 mg CO_2_-C/m^2^/hr). In contrast, lower GPP values observed in the northern part of the SE region likely reflect a combination of shorter growing seasons and more limited wetland extent.

The spatial distribution of RECO closely mirrors that of GPP, which is expected, as ecosystem respiration is strongly linked to vegetation biomass and productivity (Fig. 2). Temperature and radiation are also major controls on respiration rates, further reinforcing the similarity between the two flux patterns (Sun et al. 2023; Wei et al. 2022). The feature importance analysis supports this interpretation, showing similar rankings of the most influential predictors of GPP and RECO. EVI was the most important predictor of both fluxes in RF and XGB, highlighting the influence of vegetation. VPD was also highly important for both fluxes, while temperature, radiation, and seasonality were influential, although their relative rankings differed between fluxes and algorithms.

The NEE map (Fig. 2c) suggests that the Eastern Coast and the Everglades acts as a prominent carbon sink, with highly negative NEE values indicating that GPP exceeds RECO in this region. This finding highlights the role of dense, productive salt marsh systems such as the Everglades in driving net carbon uptake (Shoemaker et al. 2015). Our estimated sequestration rates for 2000–2020 are within order of magnitude of those reported by Song et al. (2013) for the Everglades for the 2000s (approximately 46 mg CO_2_-C m⁻^2^ hr⁻^1^, reported as positive under their sign convention), with both studies consistently identifying the region as a strong carbon sink. In contrast, interior upland areas show near-zero NEE, which may result from either lower GPP or higher respiration rates that offset potential carbon gains.

Although the spatial patterns of GPP and RECO are generally consistent, their spatial difference does not always correspond to the predicted NEE, suggesting that a carbon mass balance is not consistently preserved (Figure S17). In particular, NEE calculated as RECO − GPP shows stronger net carbon uptake than the directly predicted NEE, although localized areas of net carbon loss are also present. This discrepancy highlights an important limitation of the current ML framework, in which GPP, RECO, and NEE are modeled independently. As a result, internal carbon balance relationships are not explicitly enforced, allowing inconsistencies to emerge when fluxes are compared spatially. The difference between GPP and RECO predictions may indicate stronger net carbon uptake than those indicated by directly predicted NEE, which is directly constrained by eddy covariance observations, whereas GPP and RECO are inferred through partitioning methods. Similar challenges have been reported in previous data driven scaling up studies (Liu et al 2024; Zeng et al. 2020). This suggests that future efforts could benefit from incorporating mass balance or process informed constraints within the ML workflow.

From Fig. 2d, CH_4_ fluxes show a slight distinct spatial pattern from those of CO_2_ based fluxes. A few high-emission hotspots appear in North Carolina. These elevated CH_4_ emissions likely reflect the influence of saturated, organic-rich coastal wetlands with warm temperatures and tidal dynamics that enhance CH_4_ production and transport (Mitra et al. 2020). Despite the presence of salinity, these systems can still act as significant CH_4_ sources due to their hydrological and biological characteristics. However, it is important to note that these localized hotspots may also reflect data anomalies or uncertainties in input variables specific to this region and thus warrant further investigation. It is also important to note that our scaling up is based on a static wetland distribution, even though wetland extent can change both seasonally and over recent decades (Fluet-Chouinard et al. 2023). This limitation may affect the accuracy of long-term regional carbon estimates.

The feature importance results provide additional insight into the environmental factors associated with the predicted spatial patterns. Vegetation and climatic variables, particularly EVI, seasonality, VPD, and ${R}_{d}$, were important for predicting CO_2_ fluxes (GPP, NEE, and RECO). This result is consistent with the strong influence of vegetation activity, radiation, and temperature on photosynthesis and ecosystem respiration. In contrast, CH_4_ predictions were more strongly associated with soil and hydrological properties, including OM, Con, pH, and ${SWC}_{1}$, reflecting the importance of substrate availability and anaerobic soil conditions for methanogenesis. Con served as a proxy for salinity and, indirectly, sulfate availability in coastal wetlands, where sulfate-reducing microorganisms can outcompete methanogens for shared substrates and thereby suppress CH_4_ production (Poffenbarger et al. 2011). Although RF and XGB produced somewhat different rankings, they generally identified similar groups of influential predictors. Nevertheless, these rankings should be interpreted cautiously because correlated predictors, including climatic variables and their temporal lags, may share importance.

### Interannual trends

Although no long-term trends emerged in the annual averages of CO_2_ fluxes, our CH_4_ predictions revealed noteworthy interannual variability. Consistent with previous studies using biogeochemical and data-driven models (Saunois et al. 2025; Zhang et al. 2017; Zhu et al. 2017), our ensemble predictions also indicate slight lower CH_4_ emissions during the early 2000s (2000–2007) compared to the subsequent period of 2008–2020 (Fig. 4d).

We also compared our results to the regional CH_4_ scaling up study by He et al. (2025), which covered the southeastern U.S. from 1982 to 2010. Although their historical domain only partially overlaps with ours, both studies identified 2005 and 2010 as high emission years for CH_4_ emissions in the region (Fig. 4d). Their maximum average CH_4_ flux prediction was approximately 160 nmol CH_4_/m^2^/s, which aligning with the upper estimates from our XGB (138.8 nmol CH_4_/m^2^/s) model. However, our ANN (upper estimate around 578 nmol CH_4_/m^2^/s) model yielded substantially higher maximum annual mean, potentially indicating model sensitivity to outlier conditions or overfitting in regions with sparse data.

A key distinction of our models was the inclusion of soil properties that improved the performance of all models across all target carbon fluxes. Models trained without soil properties consistently yielded lower accuracy, highlighting the importance of including these static predictors despite their lack of temporal variability. The feature importance analysis further demonstrated that soil properties contributed considerably to the predictions, particularly for CH_4_ (Figure S7). OM was the most important predictor in XGB, while OM, Con, pH, and ${\rho}_{b}$ were influential in both RF and XGB.

Despite several differences, including their finer spatial resolution, He et al. (2025) average annual CH_4_ emission was 4.93 ± 0.11 Tg CH_4_, while in this study, it was 5.7 ± 1.7 Tg CH_4_-C. Although the estimates are relatively similar, their reported uncertainty range is narrower than ours. By accounting for inter-model variability and propagating it through the scaling up process, the ensemble-based approach captures a wider range of plausible outcomes, reflecting the high spatial and temporal variability of CH_4_ emissions in the SE US.

The annual carbon budgets also reveal that RECO (233 ± 51 Tg CO_2_-C), estimates are lower than GPP values (280 ± 38 Tg CO_2_-C), which would suggest that CO_2_ uptake exceed CO_2_ emissions in the region. Which agrees with the NEE models that produced negative values, indicating a net carbon sink and active sequestration dynamics across the SE US. To ensure consistency with eddy covariance practices, we modeled NEE directly rather than deriving it as the difference between GPP and RECO, since EC towers typically measure NEE and apply post hoc partitioning to estimate its components. Despite this, the uncertainty in NEE estimates remains high (annual average: − 20 ± 18 Tg CO_2_-C), highlighting the need for further investigation into the CO_2_ sequestration potential of wetlands in this region.

As expressed in consistent carbon units, the contribution of CH_4_ emissions is negligible compared to the net CO_2_ exchange, indicating that CO_2_ fluxes largely determine the overall carbon budget. This finding is consistent with previous large-scale assessments that reported the larger role of CO_2_ fluxes relative to CH_4_ (e.g., Bridgham et al. 2006; Zhang et al. 2002). However, because CH_4_ has a much higher warming impact per unit of carbon released than CO_2_, its role becomes more significant when the balance is framed in terms of atmospheric forcing rather than mass.

CH_4_ emissions and CO_2_ fluxes also differ fundamentally in their characteristic time scales. CH_4_ exerts a strong but short-lived climate forcing, whereas CO_2_ uptake contributes to longer-term carbon storage through soil carbon accumulation (Frolking et al. 2006). This temporal contrast highlights why interannual variability in CH_4_ emissions and long-term CO_2_ sequestration provide complementary information about wetland and carbon feedbacks.

### Limitations and future directions

Although our ensemble of ML models showed reasonable performance in predicting carbon fluxes across wetlands in the SE US, several limitations must be acknowledged. One key limitation is the use of static WL data as a predictor. It is well known that hydrological conditions are key drivers of carbon processes in wetlands, which are characterized by dynamic water level fluctuations. However, we were unable to identify temporally resolved WL data that covered our historical analysis period (2000–2020). In the feature importance analysis, WL ranked among the 10 most important predictors only for GPP (9th for RF and 4th for XGB), whereas it had relatively low importance for the other carbon fluxes. This may reflect the use of static WL data, which captured spatial differences among wetlands but not seasonal WL fluctuations that may influence carbon fluxes. Other potentially important drivers of CH_4_ dynamics, such as microbial community composition and soil oxygen availability, were also unavailable as spatially continuous datasets for the study domain and period. Although their influence could not be directly evaluated, the absence of these variables may partially explain the underestimation of the highest observed CH_4_ fluxes.

In addition to the temporal constraints, our models were also limited by their spatial resolution. While consistent with ERA5-Land, the use of 0.1° × 0.1° gridded data may be too coarse to capture fine-scale variability in wetland ecosystems. Future efforts could benefit from higher-resolution environmental datasets to improve regional scaling up. Although we aimed for consistency by sourcing all climate variables from ERA5-Land, soil properties from SSURGO, and vegetation indices from MODIS, each of these datasets carries its own inherent uncertainties and biases.

We also acknowledge the underrepresentation of coastal observation sites, which may limit model generalizability in saline or tidally influenced wetlands. These ecosystems differ significantly from freshwater wetlands due to processes such as sulfate inhibition, which is known to suppress CH_4_ emissions. Another source of uncertainty is the wetland map used to scale flux estimates. Although the NWI provides a detailed wetland inventory, uncertainties in wetland extent may influence the final flux predictions. Future studies could assess this uncertainty by comparing results derived from alternative wetland datasets, such as WAD2M, GIEMS v2.0, and GLWD, and evaluating the sensitivity of regional carbon flux estimates to wetland map selection.

Finally, the uneven distribution of observational data across sites introduced challenges for extrapolation. Our models may underperform in areas with extreme carbon flux values or in regions where the predictor variable space is poorly represented in the training data. Addressing these limitations in future research will be essential for improving the accuracy and robustness of carbon flux scaling up in wetland systems.

## Conclusions

In this study, we scaled up multi-model ensemble (MME) of GPP, RECO, NEE, and CH_4_ flux predictions across the Southeastern United States. Using site-level observations, we trained five machine learning models (Random Forest, XGBoost, Support Vector Machine, Artificial Neural Network, and Partial Least Squares Regression) with a comprehensive set of environmental drivers known to influence wetland carbon dynamics. A key novelty of this work is regional scaling up of both CO_2_ and CH_4_ fluxes within a common modeling framework that explicitly represents freshwater wetlands and saltwater ecosystems through the inclusion of a salinity proxy. Overall, the models and their ensemble average (MME) demonstrated strong performance in predicting CO_2_ fluxes, although CH_4_ predictions were less accurate. The trained models were then applied to generate spatially distributed regional flux estimates, enabling a comprehensive assessment of wetland carbon dynamics across the SE US.

The spatial patterns of the scaled up fluxes revealed distinct hotspots. Predicted GPP and RECO showed similar distributions, suggesting a strong correlation, with hotspots concentrated in the Everglades in South Florida, the Alabama-Mobile Delta, and the Mississippi River Valley, particularly the Mississippi Delta region. Our NEE predictions varied more across individual models, but the ensemble results identified Southern Florida and the Mississippi Delta as key carbon-sequestering regions. However, fully capturing their long-term carbon sequestration potential may require longer observational timescales than those considered here. CH_4_ emissions showed high variability across models, yet the same regions appeared as emission hotspots. These results underscore the dual role of wetlands as both major carbon sinks and sources of CH_4_ emissions.

All carbon fluxes exhibited clear seasonal patterns. Summer months were associated with higher productivity and greater CO_2_ and CH_4_ emissions, while fluxes declined during the winter. These seasonal cycles were strongly linked to temperature and radiation, which are key drivers of carbon exchange in wetland ecosystems. Our results indicate slightly lower average CH_4_ emissions during 2000–2007 compared to 2008–2020. The years 2005 and 2010 emerged as peak emission periods, consistent with other CH_4_ modeling studies in the region.

The final gridded product (0.1° × 0.1° resolution) spanning 2000 to 2020 provides valuable information for identifying carbon-focused management priorities and restoration opportunities in Southeastern wetlands. It can also help pinpoint under-monitored areas with high potential for carbon uptake or emission, such as the Alabama-Mobile region and the Okefenokee Swamp in northern Florida and southern Georgia. By providing spatially and temporally distributed carbon flux estimates, this work advances regional-scale assessments and helps bridge critical knowledge gaps in wetland carbon cycling.

## Supplementary Information

Supplementary material 1 DOCX (8522 KB)

## Data Availability

The datasets supporting the conclusions of this article are available in the Zenodo repository, with the national‐scale database of wetland carbon fluxes (covering the conterminous U.S. and Alaska), including all preprocessing steps, at [https://doi.org/10.5281/zenodo.21417939] and the Southeastern U.S. subset derived from the national database, together with the analysis codes used to generate the figures and results presented in this study, at [https://doi.org/10.5281/zenodo.21110601].

## Abbreviations

ANN: Artificial Neural Network
CH_4_: Methane
CO_2_: Carbon dioxide
Con: Electric Conductance
DJF: December–January–February
EC: Eddy Covariance Towers
EVI: Enhanced Vegetation Index
GPP: Gross primary productivity
JJA: June–July–August
MAE: Mean absolute error
ML: Machine learning
MME: Multi-model ensemble
NEE: Net ecosystem exchange
NSE: Nash-Sutcliffe Efficiency
NWI: National Wetlands Inventory
OM: Organic matter
PA: Atmospheric Pressure
PLSR: Partial least square regression
Prec: Precipitation
RECO: Ecosystem respiration
$${R}_{d}$$: Radiation
RF: Random Forest
RMSE: Root mean squared error
SE US: Southeastern United States
SVM: Support Vector Machine
SWC: Soil water content
VPD: Vapor Pressure Deficit
$${T}_{a}$$: Air Temperature
$${T}_{s}$$: Soil Temperature
WL: Water Level
WS: Wind Speed
XGB: EXtreme Gradient Boost
${\rho}_{b}$: Soil Bulk Density
