# Supplementary material for: Ensemble machine learning-based scaling up of wetland carbon dynamics across the Southeastern U.S
Source: Ecol Process. 2026 Oct 10;15(1):97. doi: 10.1186/s13717-026-00755-9 (PMC13653873; doi:10.1186/s13717-026-00755-9)
Supplement: Supplementary file 1 [file 13717_2026_755_MOESM1_ESM.docx]

Supporting Information

**Ensemble Machine Learning-Based Scaling up of Wetland Carbon Dynamics Across the Southeastern U.S.**

Ana Flavia Brancalion-Costaᵃ^c^, Evan Farryᵃ, Latif Kalinᵃ, Mohamed Hantushᵇ, & Zutao Ouyangᵃ

ᵃ College of Forestry, Wildlife and Environment, Auburn University, Auburn, AL 36849, USA

ᵇ U.S. EPA, Andrew W. Breidenbach Environmental Research Center, Cincinnati, OH 45268, USA

^c^ School of Resource and Environmental Management, Simon Fraser University, Burnaby, Canada

Figure S1. Mean values of gridded climate variables from ERA5-Land used as model predictors across the SE US for the period 2000–2020. Variables include: WS (m/s), Tₐ (°C), PA (kPa), Tₛ (°C), VPD (hPa), SWC (%), precipitation (mm/d), and Rd (W/m²).

Figure S2. Long-term mean EVI used as a proxy for vegetation productivity. Panel (a) shows the original MODIS Terra EVI product (MOD13A3.061) at 1 km spatial resolution. Panel (b) shows the same data regridded to 0.1° × 0.1° resolution to match the spatial scale of other environmental predictors.

**Figure S3.** Gridded maps of soil properties and water table depth (WTD) across the SE US, derived from the SSURGO database. (a) soil pH, (b) $\rho_{b}$ (g/cm³), (c) OM (%), (d) Con (dS/m), and (e) WTD (cm).

Figure S4. Regridded maps of soil properties and hydrological predictors used as input variables in the machine learning models. All layers were aggregated to a 0.1° × 0.1° resolution to match the spatial scale of other environmental predictors. Variables shown include: bulk density (g/cm³), electrical conductivity (dS/m), pH, OM (%), WL (cm; water level) and binary flooded status (1 = flooded, 0 = not flooded). Data were primarily derived from the SSURGO database and masked to wetland areas for model application.

| i) GPP | ii) RECO | iii) NEE | iv) CH_4_ |
| --- | --- | --- | --- |
| a) RF | | | |
|   **obs**  **sim** |  |  |  |
| b) XGB | | | |
|  |  |  |  |
| c) SVM | | | |
|   ←Site 13-1 |  |  |  |
| d) ANN | | | |
|  |  |  |  |
| e) PLSR | | | |
|  |  |  |  |

**Figure S5.** Scatterplots comparing predicted (y-axis) and observed (x-axis) values for the testing dataset for: (i) GPP (mg CO_2_-C m^-2^ hr^-1^), (ii) RECO (mg CO_2_-C m^-2^ hr^-1^), (iii) NEE (mg CO_2_-C m^-2^ hr^-1^), and (iv) CH_4_ (µg CH_4_-C m^-2^ hr^-1^). Each row corresponds to a different ML model: (a) RF, (b) XGB, (c) SVM, (d) ANN, and (e) PLSR. The dashed black line denotes the 1:1 reference line.

Figure S6. Empirical cumulative distribution functions (ECDFs) comparing observed and predicted CH_4_ fluxes for the (a) ensemble model, (b) Random Forest (RF), (c) Extreme Gradient Boosting (XGBoost), (d) Support Vector Machine (SVM), (e) Artificial Neural Network (ANN), and (f) Partial Least Squares Regression (PLSR). The black line represents the observed CH_4_ flux distribution, and the blue line represents the corresponding model predictions.

| a) | b) |
| --- | --- |
|  |  |

Figure S7. Feature importance of environmental predictors for wetland carbon fluxes. Heatmaps show the normalized feature importance values for predictors of GPP, RECO, NEE, and CH_4_ obtained using (a) RF and (b) XGB models. Values within the cells indicate the relative importance of each predictor, with darker colors representing greater contributions to model predictions. Importance values sum to one for each carbon flux and model.

Figure S8. Mean predicted GPP (mg CO_2_-C m^-2^ hr^-1^) across the US SE from individual machine learning models over the full study period (2000–2020). Panels show spatial averages from: RF, XGB, SVM, ANN, and PLSR.

Figure S9. Mean predicted RECO (mg CO_2_-C m^-2^ hr^-1^) across the US SE from individual machine learning models over the full study period (2000–2020). Panels show spatial averages from: RF, XGB, SVM, ANN, and PLSR. For visualization, predictions outside the 1st–99th percentile range were masked.

Figure S10. Mean predicted NEE (mg CO_2_-C m^-2^ hr^-1^) across the US SE from individual machine learning models over the full study period (2000–2020). Panels show spatial averages from: RF, XGB, SVM, ANN, and PLSR.

Figure S11. Mean predicted CH_4_ (µg CH_4_-C m^-2^ hr^-1^) across the US SE from individual machine learning models over the full study period (2000–2020). Panels show spatial averages from: RF, XGB, SVM, ANN, and PLSR. For visualization, predictions outside the 1st–99th percentile range were masked.

Figure S12. Seasonal comparison of ensemble-predicted GPP (mg CO_2_-C m^-2^ hr^-1^) across the SE US based on mean values for (left) summer months (June–August, JJA) and (right) winter months (December–February, DJF) over the period 2000–2020.

Figure S13. Seasonal comparison of ensemble-predicted RECO (mg CO_2_-C m^-2^ hr^-1^) across the SE US based on mean values for (left) summer months (June–August, JJA) and (right) winter months (December–February, DJF) over the period 2000–2020.

Figure S14. Seasonal comparison of ensemble-predicted NEE (mg CO_2_-C m^-2^ hr^-1^) across the SE US based on mean values for (left) summer months (June–August, JJA) and (right) winter months (December–February, DJF) over the period 2000–2020.

Figure S15. Seasonal comparison of ensemble-predicted CH_4_ fluxes (µg CH_4_-C m^-2^ hr^-1^) across the SE US based on mean values for (left) summer months (June–August, JJA) and (right) winter months (December–February, DJF) over the period 2000–2020.

Figure S16. Annual anomalies of climate and vegetation predictors across the SE US from 2000 to 2020. Anomalies were calculated relative to the 2000–2020 mean for TA, TS, VPD, SWC, precipitation, Rd, and EVI. Positive values indicate years above the long-term average, whereas negative values indicate below-average conditions. Precipitation anomalies were computed from annual totals, while all other variables were based on annual means.

**Figure S17.** Spatial distribution of mean NEE across wetlands in the SE US from February 2000 to December 2020. NEE was obtained (a) directly from the NEE multi-model ensemble and (b) indirectly as the difference between the RECO and GPP multi-model ensembles (NEE = RECO − GPP). Positive values indicate net CO_2_ emissions, while negative values indicate net CO_2_ uptake.

Table S1. Gridded data sources for environmental variables at the site scale.

| **Variables** | | **Gridded Data Source** | **Resolution** |
| --- | --- | --- | --- |
| **Climate** | Precipitation, $T_{a}$, VPD | PRISM - Parameter-elevation Regressions on Independent Slopes Model (https://prism.oregonstate.edu) | 4 km x 4 km |
|  | WS, $R_{d}$ | GridMET/METDATA (https://www.climatologylab.org/gridmet.html) | 4 km x 4 km |
|  | PA | NSRDB - National Solar Radiation Database (https://nsrdb.nrel.gov) | 4 km x 4 km |
|  | $T_{s}$ | ERA5-Land (https://cds.climate.copernicus.eu/datasets/reanalysis-era5-land?tab=overview) | 0.1° × 0.1° |
| **Hydrology** | SWC |  |  |
|  | WL | Soil Survey Geographic Database (SSURGO)  USDA | 1:12,000 to 1:24,000 |
| **Soil Characteristics** | pH, OM, $\rho_{b}$, Electric Conductance |  |  |
| **Vegetation** | EVI | Terra Moderate Resolution Imaging Spectroradiometer (MODIS) Vegetation Indices (MOD13A3.061) (https://modis.gsfc.nasa.gov/data/dataprod/mod13.php) | Monthly L3 Global 1 km product |

Table S2. Ranges of hyperparameters used for tuning each machine learning model during model development. The search was performed using random search and grid search strategies depending on the algorithm.

|  |  |  |  | **Best parameter** | | | | | | |
| --- | --- | --- | --- | --- | --- | --- | --- | --- | --- | --- |
| **ML** | **Parameters** | **Min** | **Max** | **GPP** | **RECO** | | | **NEE** | | **CH_4_** |
| **RF** | n_estimators | 50 | 500 | 100 | 100 | | | 500 | | 300 |
|  | max_depth | None | 50 | None | 20 | | | 10 | | None |
|  | min_samples_split | 2 | 30 | 5 | 2 | | | 5 | | 5 |
|  | min_samples_leaf | 1 | 10 | 2 | 4 | | | 2 | | 1 |
|  | max_features | [1, 'sqrt', 'log2'] | | sqrt | sqrt | | | sqrt | | log2 |
| **XGB** | n_estimators | 50 | 150 | 80 | 100 | | | 100 | | 80 |
|  | learning_rate | 0.01 | 0.2 | 0.05 | 0.07 | | | 0.075 | | 0.075 |
|  | max_depth | 2 | 6 | 2 | 3 | | | 2 | | 3 |
|  | min_child_weight | 1 | 10 | 6 | 4 | | | 6 | | 5 |
|  | gamma | 0 | 0.3 | 0 | 0.02 | | | 0 | | 0 |
|  | colsample_bytree | 0.3 | 0.7 | 0.3 | 0.6 | | | 0.2 | | 0.4 |
|  | subsample | 0.4 | 0.9 | 0.9 | 0.6 | | | 0.7 | | 0.7 |
| **SVM** | C | 0.1 | 150 | 10 | 1 | | | 1 | | 1 |
|  | epsilon | 0.01 | 1 | 0.01 | 0.1 | | | 0.5 | | 0.5 |
|  | degree | 2 | 3 | 3 | 2 | | | 3 | | 2 |
|  | coef0 | 0 | 1 | 1 | 1 | | | 1 | | 1 |
|  | gamma | ['scale', 'auto'] | | scale | scale | | | scale | | scale |
| **ANN** | model_units | 32 | 64 | 32 | 32 | | | 32 | | 64 |
|  | model_learning_rate | 0.001 | 0.01 | 0.001 | 0.001 | | | 0.001 | | 0.001 |
|  | batch_size | 16 | | | | | | | | |
|  | epochs | 200 | | | | | | | | |
| **PLSR** | n_components |  | | 2 | | 2 | 2 | | 1 | |

Table S3. Description of the wetland vegetation classes from the National Wetlands Inventory (NWI).

| **Wetland Type** | **Description** |
| --- | --- |
| **Forested** | Characterized by woody vegetation that is 6 m tall or taller. |
| **Emergent** | Characterized by erect, rooted, herbaceous hydrophytes, excluding mosses and lichens. This vegetation is present for most of the growing season in most years. These wetlands are usually dominated by perennial plants. |
| **Scrub-Shrub** | Includes areas dominated by woody vegetation less than 6 m (20 feet) tall. The species include true shrubs, young trees (saplings), and trees or shrubs that are small or stunted because of environmental conditions. |
| **Intertidal** | The substrate in these habitats is flooded and exposed by tides; includes the associated splash zone. |
